# Indirubin derivatives are potent and selective anti-*Trypanosoma cruzi* agents

**DOI:** 10.1080/21505594.2018.1532242

**Published:** 2018-11-02

**Authors:** Antonia Efstathiou, Cássio Santana Meira, Nicolas Gaboriaud-Kolar, Tanira Matutino Bastos, Vinícius Pinto Costa Rocha, Konstantina Vougogiannopoulou, Alexios-Leandros Skaltsounis, Despina Smirlis, Milena Botelho Pereira Soares

**Affiliations:** a Molecular Parasitology Lab, Dpt of Microbiology, Hellenic Pasteur Institute, Athens, Greece; b Laboratory of Tissue Engineering and Immunopharmacology, Instituto Gonçalo Moniz, Fundação Oswaldo Cruz (FIOCRUZ), Salvador, BA, Brazil; c Center of Biotechnology and Cell Therapy, Hospital São Rafael, Salvador, BA, Brazil; d Pharmacognosy and Pharmaceutical Chemistry Lab, Dpt of Pharmacy, Panepistimiopolis Zografou, University of Athens, Athens, Greece

**Keywords:** Indirubins, *T. cruzi*, antiparasitic, *in vivo* efficacy

## Abstract

Current treatment for combatting Chagas disease, a life-threatening illness caused by the kinetoplastid protozoan parasite *Trypanosoma cruzi* is inadequate, and thus the discovery of new antiparasitic compounds is of prime importance. Previous studies identified the indirubins, a class of ATP kinase inhibitors, as potent growth inhibitors of the related kinetoplastid *Leishmania*. Herein, we evaluated the inhibitory activity of a series of 69 indirubin analogues screened against *T. cruzi* trypomastigotes and intracellular amastigotes. Seven indirubins were identified as potent *T. cruzi* inhibitors (low μΜ, nM range). Cell death analysis of specific compounds [3'oxime-6-bromoindirubin(6-BIO) analogues 10, 11 and 17, bearing a bulky extension on the oxime moiety and one 7 substituted analogue 32], as evaluated by electron microscopy and flow cytometry, showed a different mode of action between compound 32 compared to the three 6-BIO oxime- substituted indirubins, suggesting that indirubins may kill the parasite by different mechanisms dependent on their substitution. Moreover, the efficacy of four compounds that show the most potent anti-parasitic effect in both trypomastigotes and intracellular amastigotes (10, 11, 17, 32), was evaluated in a mouse model of *T. cruzi* infection. Compound 11 (3ʹpiperazine-6-BIO) displayed the best *in vivo* efficacy (1/6 mortality, 94.5% blood parasitaemia reduction, 12 dpi) at a dose five times reduced over the reference drug benznidazole (20 mg/kg vs100 mg/kg). We propose 3ʹpiperazine-6-BIO as a potential lead for the development of new treatments of Chagas disease.

## Introduction

Chagas disease, or American trypanosomiasis, is endemic in 21 countries in the Central and South America and is considered the most deadly parasitic disease in Latin America, being worldwide estimated that 7–8 million people are infected with *Trypanosoma cruzi*, its causative agent [1]. The disease is increasing in other continents due to population migration. There is no human vaccine against Chagas disease, and the current chemotherapy available is based on nifurtimox or benznidazole [2]. These drugs show good efficacy when given during the acute phase, up to 2 to 6 months after infection. About 30% of the chronic cases will develop cardiovascular disease, while 10% will manifest neurological or/and gastrointestinal problems [3]. As there are serious drawbacks of current treatments, especially their toxicity, new effective treatments for this disease are urgently needed.

Indirubin is a vivid red compound found in plants and molluscs. It is a minor ingredient of indigo dye derived from plants (*Isatis spp., Indigofera spp., Polygonum spp*.), and the infamous Tyrian purple prepared since antiquity from molluscs of the family Muricidae [4]. In addition, indirubin as well as indigo are produced as a result of bacterial metabolism, a phenomenon that is evident in the clinical condition of Purple Urine Bag Syndrome (PUBS), where the urine of patients with a urinary tract infection turn purple [5]. More importantly, indirubin was found to be the active constituent of the Traditional Chinese Medicine preparation Danggui Longhui Wan, used to treat leukemia [4].

The basic indirubin scaffold can be modified to generate analogue compounds by different synthetic and therapeutic processes [6]. Indirubins have shown to possess several biological properties, including anticancer [7–9], antiviral [10,11], as stem cell regulators [12,13], neuroprotective [14,15] and anti-inflammatory agent for psoriasis [16] and endotoxemia [17] by targeting different kinases such as glycogen synthase kinase 3 (GSK3), cyclin dependent kinases (CDKs) and dual-specificity tyrosine-regulated kinases (DYRKs) [18–21]. Additionally, they possess antiparasitic activity against the trypanosomatids *Leishmania* and *Trypanosoma brucei*, by inhibiting kinases, including *Leishmania* (*L*) glycogen synthase kinase 3 short (GSK-3s) [22] and *T. brucei (Tb)* GSK-3s kinases [23], respectively.

Since *T. cruzi* belongs to the same family of trypanosomatids, such as *Leishmania* and *T. brucei* [24], in this study we performed a screening to identify novel *T. cruzi* inhibitors amongst 69 compounds of an *in house* indirubin collection (Table S1). The identified inhibitors were further studied to evaluate their mode of action by determining the mechanism of cell death using electron microscopy and FACS analysis. The results obtained highlighted the different mode of action between the 6- and 7- substituted indirubins. Finally, the efficacy of selected indirubins was evaluated in a murine model of *T. cruzi* infection.

## Results

### Trypanocidal and cytotoxic activities

The trypanocidal activity of the 69 indirubins was initially evaluated against trypomastigotes (Y strain *T. cruzi*) through the determination of EC_50_ values. First, the collection of 69 indirubins (Table S1) was screened against the trypomastigote form of *T. cruzi* parasites at 10 μΜ and 1 μΜ. In the initial screening, only 7 compounds were found to cause at least 50% growth inhibition in *T. cruzi* parasites at both concentrations tested (data not shown) and subsequently their EC_50_ values was determined. Thus, 7 out of the 69 compounds showed a potent anti*-T. cruzi* activity, being able to kill trypomastigotes with EC_50_ values ranging from 0.25 to 1.3 μM (compounds 5, 10–13, 17 and 32, Table 1). The standard drug benznidazole presented an EC_50_ of 11.3 µM. The 7 identified compounds active against *T. cruzi* trypomastigotes included six 3’substituted 6-BIO (5, 10–13 and 17) and one 7-substituted (32) indirubins. Compounds 5, 12 and 13 displayed high cytotoxicity against murine macrophages J774.1 (Table 1). In contrast, compounds 10, 11, 17 and 32, which had lower cytotoxicity against macrophages, were further investigated for their anti-trypanosomal activity against the intracellular amastigote form of the parasites. Importantly, the EC_50_ values for the amastigotes were also in the low micromolar/nanomolar range, between 0.22–1.51 μΜ and compounds displaying selectivity index (SI) values above 6.6 (Table 1; Figure S1).

**Table 1.** Cytotoxicity against murine macrophages J774.1 and anti-*Trypanosoma cruzi* activity against trypomastigote and amastigote forms of indirubins.

Compound	EC_50_ Try. (µM)^a^	CC_50_ (µM)^b^	EC_50_ Ama. (µM)^c^	SI^d^
Compound 10	0.7 (± 0.2)	> 10	0.61 (± 0.14)	> 16
Compound 11	0.25 (± 0.05)	6.25	0.22 (± 0.11)	28
Compound 12	0.49 (± 0.2)	2.6	-	-
Compound 13	0.45 (± 0.16)	2.4	-	-
Compound 17	0.44 (± 0.14)	10	0.90 (± 0.24)	11
Compound 5	0.37 (± 0.16)	1.5	-	-
Compound 32	1.30 (± 0.46)	> 10	1.51 (± 0.45)	> 6.6
Benznidazole	11.4 (± 1.4)	> 100	13.4 (± 0.39)	> 7.5

CC_50_ = cytotoxicity concentration 50 %. EC_50_ = effective concentration 50 %. SI = Selective index. Values are means ± SD of three independent experiments performed in triplicate.

^a^Trypanocidal effect on trypomastigotes forms determined 24 h post-treatment.

^b^Cell viability of murine macrophages J774.1 determined 72 h post-treatment.

^c^Trypanocidal effect on amastigotes forms determined in infected macrophages treated for 6 h.

^d^Calculated by dividing the CC_50_/EC_50_ Ama.

### Investigating the mechanisms of action of indirubins

**Figure 1.**
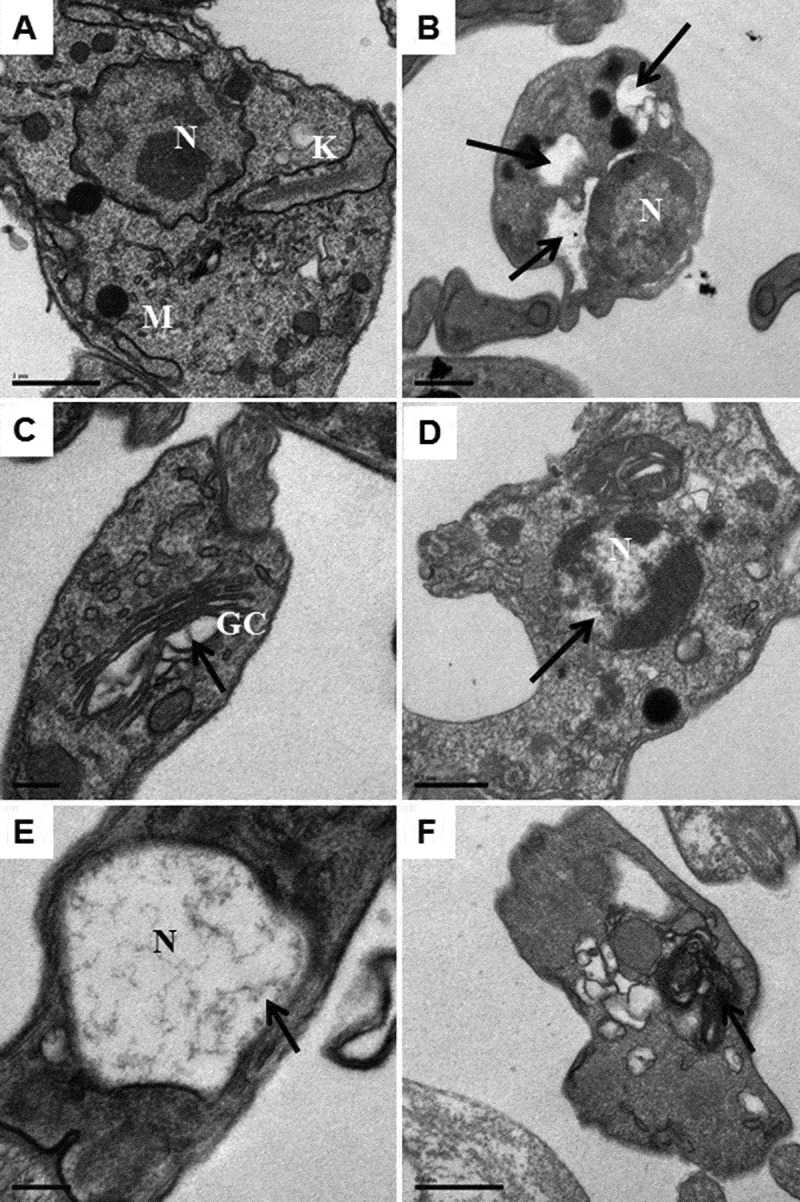
Transmission electron microscopy of *T. cruzi* trypomastigotes incubated with indirubins for 24 h. (a) Untreated cells, scale bar: 1 μm. (B-F) After incubation with indirubins, we observed: (b) loss of cytoplasm after incubation with the EC_50_ concentration of the compound 10, scale bar: 0.5 μΜ, (c) dilatation of some Golgi cisternae after treatment with the EC_50_ concentration of the compound 32, scale bar: 0.2 μm, (d) DNA degradation with pyknotic nuclei after incubation with the EC_50_ concentration of the compound **11**, scale bar: 0.5 μΜ, (e) DNA degradation with pyknotic nuclei after incubation with the EC_50_ concentration of the compound 17, scale bar: 0.2 μΜ and (f) appearance of myelin figures after incubation with the EC_50_ concentration of the compounds 10, 11, 17 and 32, scale bar: 0.5 μm. Black arrows indicate changes in the organelles.

Transmission electron microscopy (TEM) was used to examine the ultrastructural alterations caused by the treatment with the indirubins 10, 11, 17 and 32. Thin sections of untreated trypomastigotes observed by TEM revealed normal appearance of organelles, intact plasma membrane and cytoplasm without alterations (Figure 1(a)). Treatment with 6-substituted indirubins 10, 11 and 17, however, caused loss of cytoplasm (Figure 1(b)), resulted in pyknotic nuclei displaying DNA degradation (Figure 1(d,e)) and appearance of myelin figures (Figure 1(f)). Similar alterations were observed after the treatment with the 7-substituted indirubin (32), which also caused a dilatation of Golgi cisternae (Figure 1(c)).

**Figure 2.**
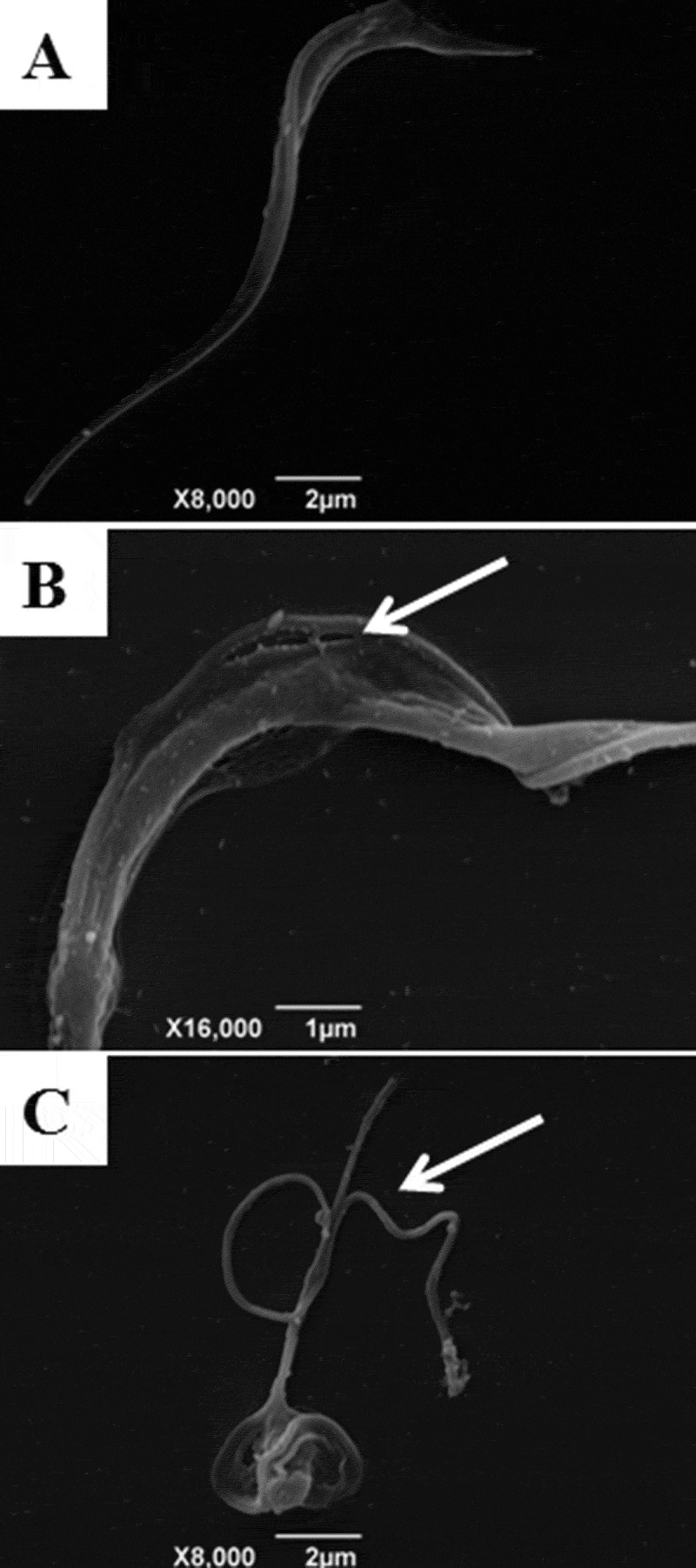
Scanning electron microscopy of *T. cruzi* trypomastigotes incubated with indirubins for 24 h. Using SEM, we observed alterations in cell shape and loss of plasma membrane integrity after treatment with the EC_50_ and 2 x EC_50_ concentration of compound 11 (B and C respectively) and (A) untreated cells. White arrows indicate the alterations reported.

Scanning electron microscopy (SEM) was also employed to study the morphology of trypomastigotes treated or not with indirubins 10, 11, 17 and 32. Untreated trypomastigotes had the typical elongated shape of the parasite without visible alterations in the plasma membrane or in cell volume (Figure 2(a)). Trypomastigotes treated with 6-substituted indirubins 10, 11 and 17 and 7-substituted indirubin 32 displayed extensive loss of plasma membrane integrity. Representative images of trypomastigotes treated with the most potent compound herein, compound 11, at 0.25 and 0.5 µM for 24 h, are shown in Figure 2(b,c), respectively.

**Figure 3.**
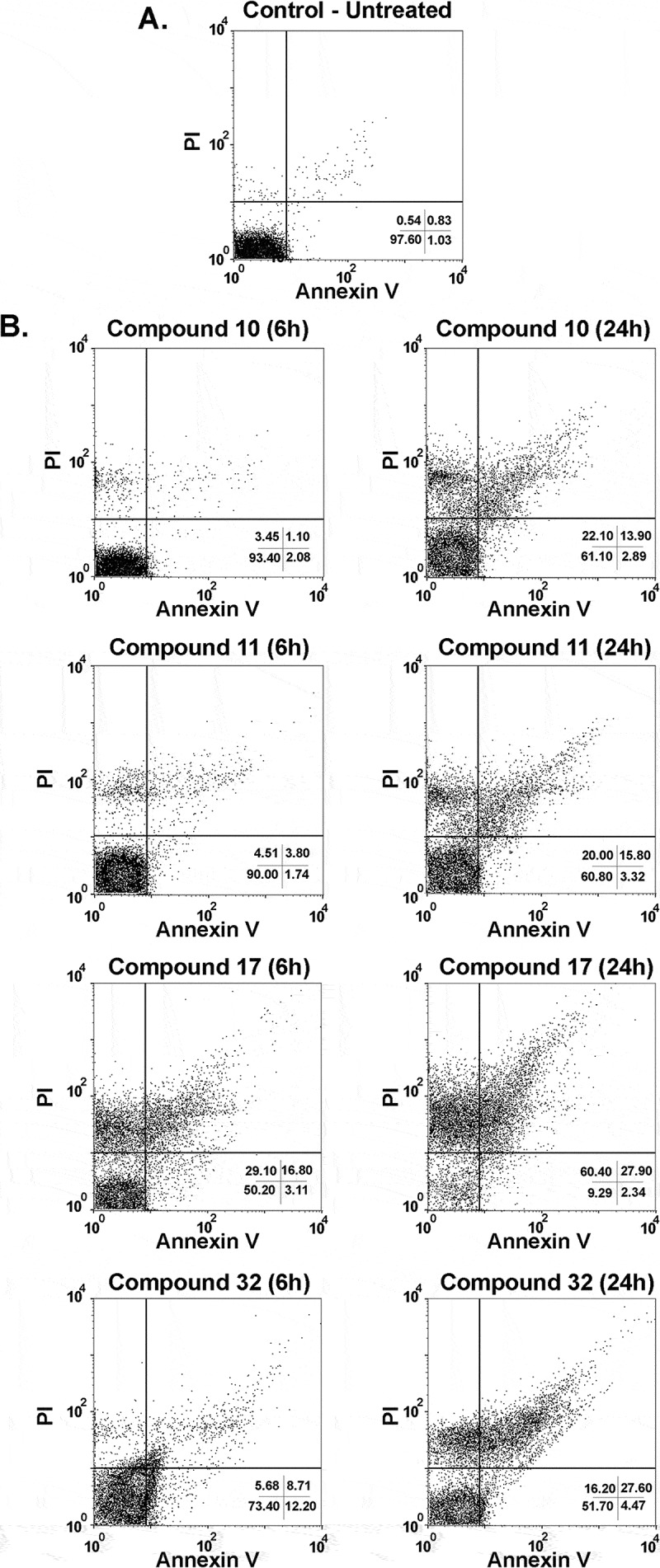
Flow cytometry analysis of trypomastigotes treated with indirubins 10, 11, 17 and 32 at the EC_50_ concentration, upon propidium iodide (PI) and annexin-V staining. (A) Untreated trypomastigotes after 24 h of incubation; (B) trypomastigotes incubated with indirubins 10, 11, 17 and 32 for 6 and 24 h.

Next, to further understand the mechanism by which indirubins cause parasite death in *T. cruzi*, a double staining with annexin V and propidium iodide (PI) was performed in indirubin-treated parasites for 6 and 24 h followed by flow cytometry analysis. In untreated cultures, 97.6% of the cells were viable (annexin V-PI negative) (Figure 3(a)). Cultures treated with the EC_50_ concentration of 6-substituted indirubins showed an immediate increase in the number of double positive (apoptotic-like cells) and annexin V-negative, PI-positive (necrotic cells) populations after 6 h of treatment for indirubin 17 and after 24 h for indirubins 10, 11 and 17, without the appearance of early apoptotic-like parasites (annexin V positive-PI negative) (Figure 3). These results indicate that 6-substituted indirubin treatment probably causes death by a necrotic process. On the other hand, treatment with the 7-substituted indirubin (32) caused an apoptotic-like cell death after 6 h of treatment, when we observed an increase of the early apoptotic population (12.2%), which then at 24 h progresses to late-apoptotic like and necrotic populations (27.6% and 16.2%, respectively) (Figure 3).

### *In vivo* experiments

**Figure 4.**
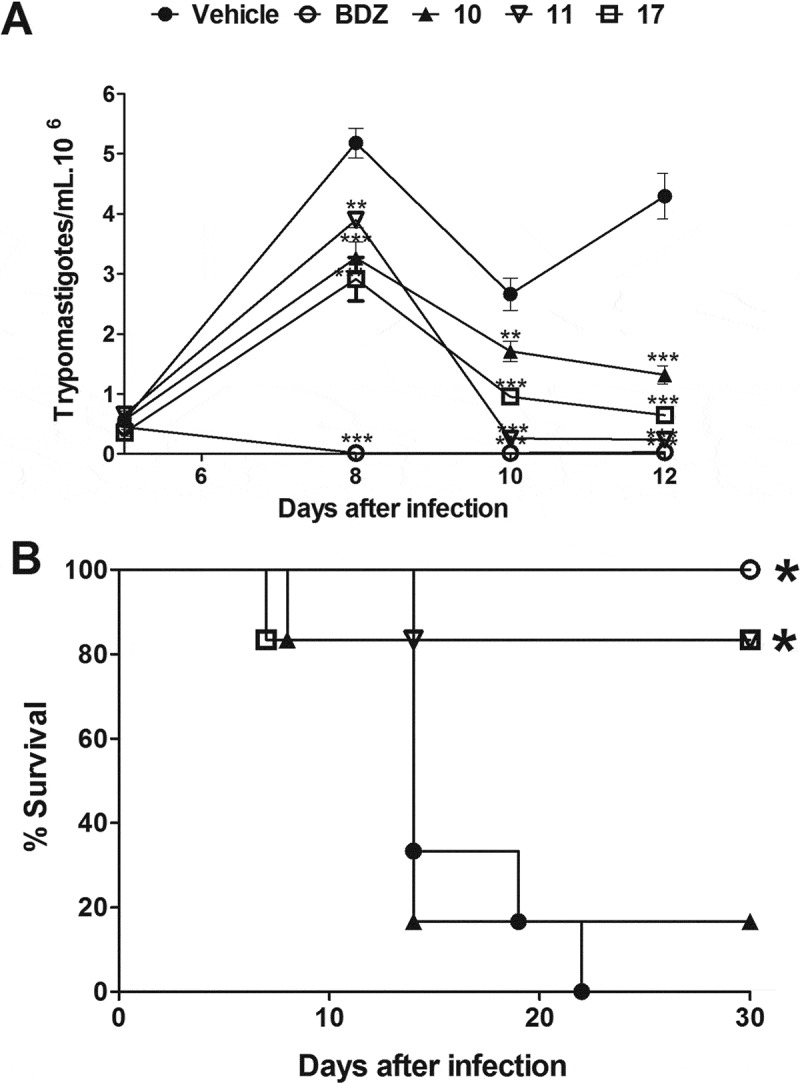
Parasitemia and survival rate of BALB/c mice infected with *T. cruzi* and treated with 6-substituted indirubins. Female BALB/c mice were infected with 10^4^ Y strain trypomastigotes. Five days after infection, mice (n = 6) were treated orally with indirubins 10, 11 and 17 (20 mg/Kg) or benznidazole (100 mg/kg) daily, during five consecutive days. (A) Parasitemia was monitored by counting the number of trypomastigotes in fresh blood samples. Values represent the mean± SEM of 6 mice per group. (B) Survival was monitored by 30 days after infection. Results are from one experiment of two performed. ** P < 0.01; *** P < 0.001 compared to untreated-infected group (vehicle).

**Figure 5.**
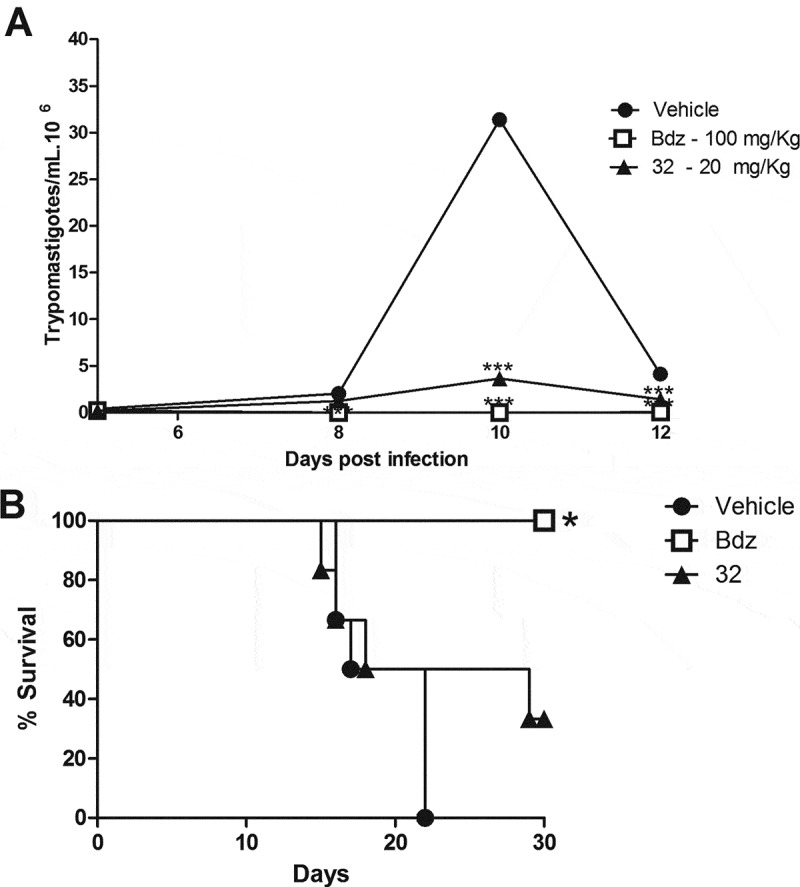
Parasitemia and survival rate of BALB/c mice infected with *T. cruzi* and treated with 7-substituted indirubin. Female BALB/c mice were infected with 10^4^ Y strain trypomastigotes. Five days after infection, mice (n = 6) were treated orally with indirubin 32 (20 mg/Kg) or benznidazole (100 mg/kg) daily, during five consecutive days. (A) Parasitemia was monitored by counting the number of trypomastigotes in fresh blood samples. Values represent the mean± SEM of 6 mice per group. (B) Survival was monitored by 30 days after infection. Results are from one experiment of two performed. ** P < 0.01; *** P < 0.001 compared to untreated-infected group (vehicle).

Next, the *in vivo* efficacy of the indirubins 10, 11, 17 and 32 was evaluated in a mouse model of acute Chagas disease. Indirubin doses of 10 or 20 mg/Kg were selected according to previous dosing regiments of other indirubin backbone analogues in mice [25], and presented no signs of toxicity or mortality. More specifically, no mortality was observed during the treatment and for up to 3 months post treatment with indirubins tested at doses of 10 or 20 mg/Kg for 5 consecutive days. Mice exhibited normal behavior, good appetite and no weight loss or external signs of fatigue. Compound 11, that exhibited higher activity against macrophages (J774.1) *in vitro*, was further examined *in vivo* by evaluating the serum and urine levels of renal and hepatic function markers (Table S2), in which mice treated with this compound showed no signs of toxicity. Thus, the highest dose was selected (20 mg/kg) and administrated, as described in material and methods section. As shown in Figure 4(a), all the 6-substituted indirubins 10, 11 and 17 tested significantly (*P* < 0.001) reduced blood parasitaemia (at a rate of 69.3%, 94.5% and 85%, respectively, after 12 days post infection – Table 2) when compared to mice treated with an equal volume of vehicle (DMSO-PBS) (Figure 4(a)). The 7- substituted indirubin, compound 32 was also shown to reduce parasitaemia but at a rate of 34.63% after 12 days post infection (Figure 5(a), Table 2). Treatment with benznidazole, the standard drug, at 100 mg/kg dose resulted in > 99% of inhibition of blood parasitaemia. Treatment with indirubins 11 and 17, in contrast to the indirubin 10 and 32, similar to the treatment with benznidazole, had a protective effect, reducing mortality (Table 2).

**Table 2.** Parasitemia and mortality in mice infected with Y *Trypanosoma cruzi* strain and treated daily with 20 mg/Kg of indirubins or 100 mg/Kg of benznidazole for 5 days.

Compounds	Dose (mg/Kg)	% Blood parasitemia reduction in mice at 12 dpi^a^	Mortality^b^
Compound 10	20	69.3%*	5/6
Compound 11	20	94.5%*	1/6*
Compound 17	20	85%*	1/6*
Compound 32	20	34.63*	4/6
Benznidazole	100	> 99*	0/6*
Vehicle		-	6/6

^a^Calculated as ([average vehicle group − average treated group)/average vehicle group] × 100%).

^b^Mortality was monitored for 30 days after treatment. Bdz = benznidazole. *P < 0.05 compared to vehicle group.

## Discussion

In previous studies, indirubins had been evaluated against a variety of diseases particularly in cancer and neurodegenerative disease therapy studies [8,26–28] and have been shown to be potent inhibitors of mammalian kinases such as CDKs, GSK-3β, DYRKs and Aurora kinases [4,6,18,29]. Recently, indirubins have also drawn the attention of scientific groups working on antiparasitic drug discovery [22,23,30,31], highlighting their potential as chemotherapeutic agents against the kinetoplastid protozoans *L. donovani* [22,30] and *T. brucei* [23]. Indirubins have been shown to target parasitic kinases, such as GSK-3 [22,23,30], CRK3 (homologue of the mammalian CDK1) [22,30,32] and casein kinase 1 (CK1) [31].

The sequence homology of kinetoplastid kinases and the inhibitory action of indirubins on the activity of specific *Leishmania* and *T. brucei* kinases [23] prompted us to further evaluate the antiparasitic activity of a 69-indirubin in-house collection against *T. cruzi*. Herein, our results showed that the indirubin compounds were effective inhibitors of the growth of *T. cruzi* parasites. Surprisingly, only 7 out of the 69 compounds showed a potent anti-*T. cruzi* activity, compared to 13 and 32 out of 69 that previously showed inhibitory activity against *Leishmania* and *T. brucei*, respectively [22,23,30]. It is noteworthy that some of the most potent inhibitors of *Leishmania* and *T. brucei* parasites [22,23,30], namely 6BIO and 5-Me-6BIO, did not exhibit any activity against the screening threshold of 10 μΜ in *T. cruzi* trypomastigotes. The latter observation could be due to: a) different intake of indirubins, b) different levels of expression of the proteins targeted by the compounds in the three parasites, c) different role of the protein targets between the three parasites, d) different affinity of the compounds in the ATP binding pocket of the protein-target or/and e) different protein targets in the three parasites. The identified indirubins that herein act as inhibitors of the *T. cruzi* trypomastigotes, however, are also potent inhibitors of *Leishmania* and *T. brucei* parasites and could serve as scaffolds for anti-kinetoplastid drug discovery.

In our study, four out of seven compounds (indirubins 10, 11, 17 and 32) were identified as inhibitors of both *T. cruzi* trypomastigotes and intracellular amastigotes, while having good selectivity against mammalian cells. Those compounds display lower EC_50_ values (EC_50_Try: 0.25–1.30 μΜ, EC_50_Ama: 0.22–1.51 μΜ) than the standard drug benznidazole (EC_50_ of 11.4 and 13.4 µM for trypomastigotes and intracellular amastigotes upon 24 h and 6 h of treatment, respectively) *in vitro*, which implies that they are potent inhibitors of the *T. cruzi* growth. In the literature, benznidazole is reported to inhibit *T. cruzi* amastigotes *in vitro* with EC_50_ values ranging from 1–15 μΜ, this variation being the result of the use of different host cell types, strains of parasites used and time of exposure to test compounds [33–35]. Compounds 10, 11 and 17 are 3’substituted-6BIO analogues and were identified as most potent inhibitors of *Leishmania* and *T. brucei* parasites, as well as of their parasitic kinase-targets (GKS-3s, CRK3) [22,23], while compound 32 is a 7-substituted indirubin. The 6-substituted compounds target *T. cruzi* trypomastigotes and amastigotes with EC_50_ values: 0.22–0.9 μΜ, while the active 7-substituted indirubin (compound 32) with an EC_50_ value 1.30 μΜ. As evidence exists that 3’substituted-6BIO analogues target more efficiently the CRKs and GSK-3 kinases in *Leishmania*, while 7-substituted are less potent inhibitors and are known inhibitors of human DYRKs and Aurora kinases [18], it is possible that these selected indirubins act by inhibiting different kinases.

Differences in potency among the selected compounds were also correlated to differences in the mode of death. While all the 4 selected indirubins, namely compounds 10, 11, 17 and 32, caused the loss of the parasitic plasma membrane integrity and cytoplasm, DNA degradation with pyknotic nuclei and the appearance of myelin figures, only the 7-substituted indirubin 32 caused dilatation of Golgi cisternae. Thus, our results are suggestive of a supplementary or different mode of action or target of this compound against *T. cruzi* parasites.

Further investigation with the use of flow cytometry confirmed that parasitic death by 3’substituted-6BIO analogues, results in necrosis as parasites displayed rapid cell permeability and cytoplasmic swelling, associated frequently with necrotic parasitic death [36,37]. Similar results can be found when *T. cruzi* is treated with benznidazole [38], whose action is strictly associated with oxidative stress induction in parasites [39]. On the other hand, compound 32 causes an apoptotic-like cell death during the first 6 h of treatment, as there is an increase in the early apoptotic population (14.5%), which later on (24 h) gives rise to late-apoptotic like and necrotic populations (17.1% and 13.8%, respectively). What is worth mentioning is that the mode of death induced by 3’substituted-6BIO in *T. cruzi* is different from the mode of death caused by the same compounds against both *Leishmania* and *T. brucei* [22,23]. Conceivably, treatment of *Leishmania* and *T. brucei* by EC_50_ concentrations of 3’substituted-6BIO derivatives, causes apoptosis-like death, a term used here to describe the phenotype. The latter observation reinforces the suggestion that in *T. cruzi* there is a different target or mode of action.

Finally, through the *in vivo* test for the four compounds in the acute phase of the mouse model of *T. cruzi* infection, the 3ʹpiperazine-6BIO (compound 11) emerged as a promising scaffold for drug discovery against Chagas disease, as it reduced blood parasitaemia at a rate 94.5% after 12 days of infection and it had a protective effect on mortality rate (80% survival rate). It is interesting to note that although compounds 10 and 11 are the base and salt of the same structure, a large difference is observed in terms of the inducing mortality rate. The calculated logD for compound 10 [20] is −0.87, suggesting that water solubility of this compound is probably high while lipophilicity is low, and therefore the permeability is expected to be reduced [40]. In fact, treatment with 3ʹpiperazine-6BIO (compound 11) reduced blood parasitaemia (94.5%) and increase survival rate (80%) at a similar rate to benznidazole (100% survival rate), the current drug for Chagas disease, when tested in a dose of 5X lower than that of benznidazole. The benefits observed after the treatment with compound 11 in a short-term therapeutic scheme may be increased in future investigations where treatment for 20 consecutive days will be performed in the acute phase of the disease, instead of only 5 consecutive days. Moreover, compound 11 will be also tested against the chronic phase of Chagas disease in an animal model. As 3ʹpiperazine-6BIO (compound 11) is one of the most potent inhibitors against both *Leishmania* and *T. brucei* parasites, it may be used as a scaffold for future antikinetoplastid drug discovery. Combination of drugs is the new trend of combating parasitic diseases in order to prevent resistance of the therapy [41,42] and thus compound 11 could also serve in the future as a scaffold for designing a combinatorial therapy with benznidazole for treating Chagas disease. 3ʹpyrrolidine-6BIO (compound 17) also reduced blood parasitaemia (85%) and increase survival rate (80%) at a significant rate related to benznidazole (100% survival rate), when tested in a dose of 5X lower than that of benznidazole, which implies that the insertion of a ring (piperazine or pyrrolidine) at position 3ʹ of 6BIO could lead to more effective inhibition of *T. cruzi* parasites. The latter observation is in accordance to literature, where the insertion of a ring (piperazine or pyrrolidine) at position 3ʹ of 6BIO led to increased activity against *Leishmania* parasites *in vitro* by inhibiting more specifically leishmanial Glycogen Synthase Kinase-3short (*L*GSK3s) [22]. Compound 32, a 7-substituted indirubin, presented a low inhibition of blood parasitaemia after 12 days of infection and it had a weak protective effect on mortality rate.

Altogether, these findings reinforce that indirubins are potent and selective trypanocidal agents, especially 3’substituted-6BIO analogues. Therefore, the screening for structurally-related indirubins derivative for Chagas disease treatment is an attractive line of drug development.

## Materials and methods

### Drugs

Indirubin derivatives (Table S1) were synthesized from isatin, as previously described [19,20,43,44]. Purity of the tested compounds was > 98%, and determined by means of HPLC-DAD. Benznidazole (LAFEPE) was used as a reference anti-*T. cruzi* drug. All compounds were dissolved in DMSO (Sigma-Aldrich; Catalog number: W387520) and diluted in cell culture medium for use in the assays. The final concentration of DMSO was less than 1% in all *in vitro* experiments and less than 20% in all *in vivo* experiments.

### Parasites

The *T. cruzi* Y strain (DTU II) [45] was used in this study. Tissue culture trypomastigotes were obtained from the supernatants of 5 to 6-day-old infected LLC-MK2 cells maintained in Dulbecco’s modified Eagle medium (DMEM; Catalog number: 11,965–084) supplemented with 10% fetal bovine serum (FBS; GIBCO) and 50 µg/mL of gentamycin (ThermoFisher Scientific, Catalog number: 15,750,060) at 37°C in a 5% humidified CO_2_ atmosphere. For *in vivo* analysis, bloodstream trypomastigotes used were obtained from BALB/c mice previously infected intraperitoneally with 10,000 Y trypomastigotes, and then bled after 8–12 days when they reached the peak of parasitaemia.

### Animals

Female BALB/c mice (18–22 g) were provided by the animal breeding facility and maintained in sterilized cages under a controlled environment, receiving water *ad libitum* and a balanced diet for rodent at Instituto Gonçalo Moniz (Fundação Oswaldo Cruz, Bahia, Brazil).

### Assessment of cytotoxicity to mammalian cells

Mouse macrophages (J774.1 cell line) were seeded into 96-well plates at a cell number 1 × 10^4^ cells/well in DMEM medium supplemented with 10 % of FBS and incubated for 24 h at 37°C and 5% CO_2_. Samples were then added at eight concentrations (ranging from 0.078 to 10 µM) in triplicate, and incubated for 72 h, followed by the addition of 20 µL/well of Alamar Blue (Invitrogen; Catalog number: DAL1025). Colorimetric readings were performed 24 h later at 570 and 600 nm. CC_50_ values were calculated using data-points gathered from three independent experiments. Benznidazole was tested in concentrations ranging from 0.78 to 100 µM.

### Cytotoxicity for trypomastigotes

Trypomastigotes (4x10^5^ cells/well) were seeded into 96-well plates and the indirubins were added at eight concentrations ranging from 0.08 to 10 µM, in triplicate. Plates were incubated for 24 h at 37ºC and 5% of CO_2_. Aliquots of each well were collected and the number of viable parasites, based on parasite integrity and motility, was assessed in a Neubauer chamber and compared to untreated cultures. Three independent experiments were performed.

### *In vitro* infection assay

Peritoneal exudate macrophages were obtained by washing, with cold PBS, the peritoneal cavity of BALB/c mice 4–5 days after injection of 3% thioglycolate (Sigma-Aldrich) in saline (1.5 mL per mice). Then, cells were plated at a cell density of 2 × 10^5^ cells/well in 24-well plates with sterile coverslips on the bottom in RPMI supplemented with 10% FBS and incubated for 24 h at 37° C and 5% CO_2_. Cells were then infected with trypomastigotes at a ratio of 10 parasites per macrophage for 2 h. Free trypomastigotes were removed by successive washes using saline solution. Cultures were incubated in complete medium alone or with the compounds under investigation in different concentrations for 6 h. The medium was replaced with fresh medium and the plate was incubated for 3 days. Cells were fixed in absolute alcohol and the percentage of infected macrophages and the mean number of amastigotes/100 macrophages was determined by manual counting after hematoxylin and eosin staining in an optical microscope (Olympus). The percentage of infected macrophages and the relative number of amastigotes per macrophage was determined by counting 100 cells per slide.

### Ultrastructural studies

Trypomastigotes at a cell density of 1 × 10^7^ cells/mL in 24 well-plates were treated with indirubins at EC_50_ value, twice EC_50_ value or not for 24 h. Parasites were then fixed with 2% formaldehyde and 2.5% glutaraldehyde in sodium cacodylate buffer (0.1 M, pH 7.2) for 1 h at room temperature. After fixation, parasites were washed 3 times with sodium cacodylate buffer (0.1 M, pH 7.2), and post-fixed with a 1.0 % solution of osmium tetroxide containing 0.8% potassium ferrocyanide (Sigma-Aldrich; Catalog number: P3289) for 1 h. Cells were subsequently dehydrated in increasing concentrations of acetone (30, 50, 70, 90 and 100%) for 10 min at each step and embedded in polybed resin (PolyScience; Catalog number: 00552–500). Ultrathin sections on copper grids were contrasted with uranyl acetate and lead citrate and observed under a ZEISS 109 transmission electron microscope. For scanning electron microscopy, trypomastigotes treated and fixed in the same conditions were washed in 0.1 M cacodylate buffer, and allowed to adhere in coverslips pre-coated with poly-L-lysine (Sigma-Aldrich; Catalog number: P4707). Cells were then post-fixed with a solution of osmium tetroxide containing 0.8% of potassium ferrocyanide for 30 min and dehydrated in crescent concentrations of ethanol (30, 50, 70, 90 and 100%). The samples were dried until the critical point, metallized with gold and analyzed in a JEOL JSM-6390LV scanning electron microscope. Two independent experiments were performed.

### Propidium iodide and annexin v staining

*T. cruzi* Y strain trypomastigotes (1x10^7^) were incubated for 6 and 24 h at 37 °C in the absence or presence of indirubins (EC_50_ values). After incubation, the parasites were labeled for propidium iodide (PI) and annexin V using the annexin V-FITC apoptosis detection kit (Sigma-Aldrich; Catalog number: APOAF-20TST), according to the manufacturer’s instructions. Acquisition was performed using a BD FACS Calibur flow cytometer (Becton Dickinson) and data were analyzed in BD CellQuest software. A total of 10,000 events were acquired in the region previously established as that corresponding to trypomastigotes forms of *T. cruzi*. Three independent experiments were performed.

### Infection in mice

Female BALB/c mice (18–22 g) were infected with bloodstream trypomastigotes by intraperitoneal inoculation of 10^4^ parasites in 100 µL of saline solution and then mice were randomly divided in groups (six animals per group). Before starting the treatment on the fifth day post infection, the infection was confirmed in all animals where on average 0.4 × 10^6^ parasites per ml of blood were found. Thus, after day 5 of infection, treatment with 20 mg/kg weight of indirubins 10, 11, 17 and 32 were given orally for five consecutive days. For the control group, benznidazole was given orally at dose of 100 mg/kg weight. Saline containing 20% DMSO was used as a vehicle and administrated on untreated and infected group. Infection was monitored daily by counting the number of motile parasites in 5 µL of fresh blood sample drawn from the lateral tail veins, as recommended by standard protocol [46]. Survival of mice was monitored for 30 days after treatment.

### Statistical analyses

To determine the cytotoxicity concentration 50% of J774.1 (CC_50_) and the effective concentration 50% (EC_50_) of the trypomastigotes and amastigotes forms of *T. cruzi*, we used nonlinear regression. The selectivity index (SI) was defined as the ratio of CC_50_ by EC_50_ (trypomastigotes). The one-way ANOVA followed by Bonferroni’s multiple comparison test was used to determine the statistical significance of the group comparisons in the *in vitro* infection and *in vivo* studies. Results were considered statistically significant when *P *< 0.05. All analyzes were performed using Graph Pad Prism version 5.01

### Ethics statement

All *in vivo* experiments were carried out in accordance with the recommendations of Ethical Issues Guidelines and were approved by the local Animal Ethics Committee (Approved number: L-IGM-016/2013)
