# Supplementary material for: Indirubin derivatives are potent and selective anti-Trypanosoma cruzi agents
Source: Virulence. 2018 Nov 2;9(1):1658–68. doi: 10.1080/21505594.2018.1532242 (PMC7000199; doi:10.1080/21505594.2018.1532242)
Supplement: Supplemental Material [file kvir-09-01-1532242-g000.zip › Table S1.docx]

**Table S1. Complete list of the indirubin analogues screened against *T. cruzi* parasites**

|   Indirubin backbone showing substitutions at positions 3’, 5, 6, and N1 (Y-R5, R6, R1 and R7 respectively) | | | | | | | | | **EC_50_ in *T.cruzi***  **trypomastigotes**  **(μΜ)** |  |
| --- | --- | --- | --- | --- | --- | --- | --- | --- | --- | --- |
| **Cpd** | **Y** | **R_1_** | **R_2_** | **R_3_** | **R_4_** | **R_5_** | **R6** | **R7** |  |  |
| **1** | NO | Br | H | H | H | **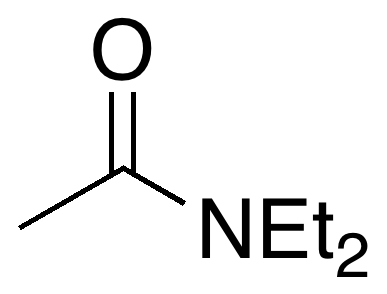** | H | H | >10 | **6-BIO -3’ bulky substituted** |
| **2** | NO | Br | H | H | H | **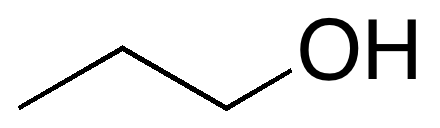** | H | H | >10 |  |
| **3** | NO | Br | H | H | H | **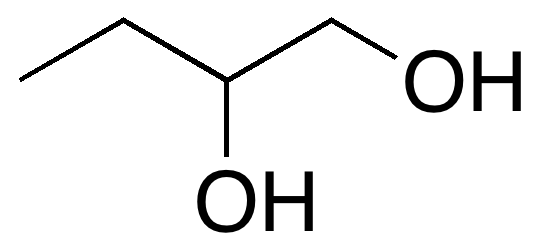** | H | H | >10 |  |
| **4** | NO | Br | H | H | H | **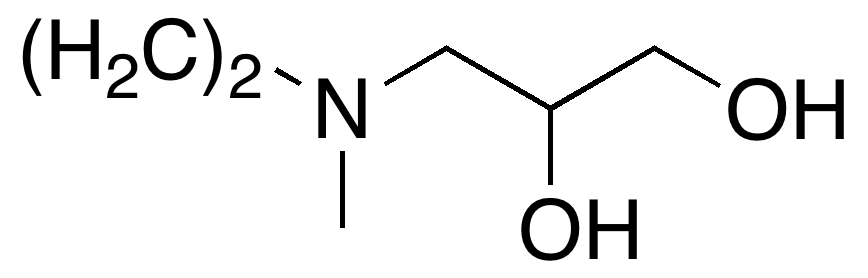** | H | H | >10 |  |
| **5** | NO | Br | H | H | H | **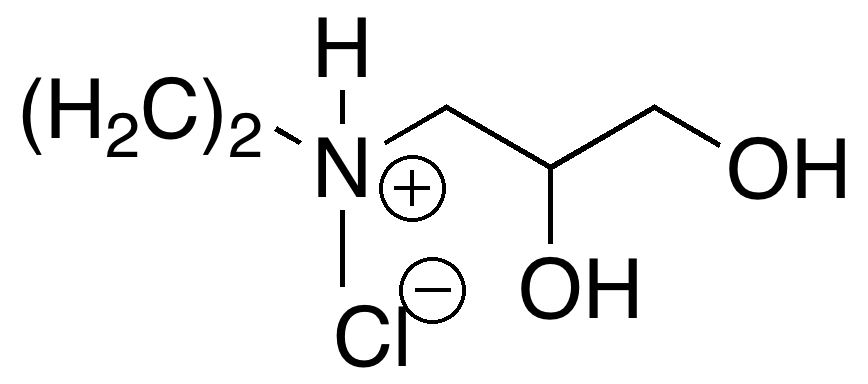** | H | H | 0.37 (± 0.16) |  |
| **6** | NO | Br | H | H | H | **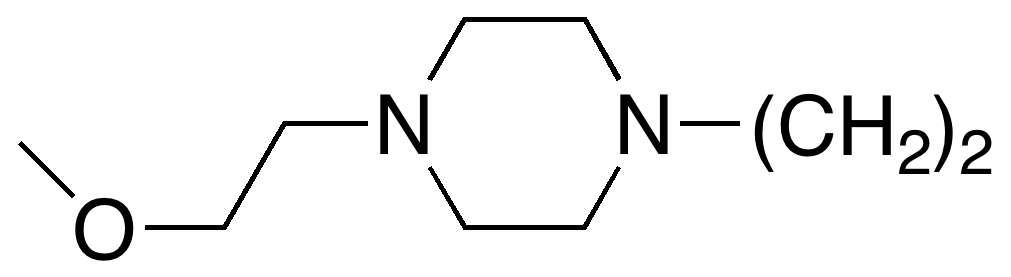** | H | H | >10 |  |
| **7** | NO | Br | H | H | H | **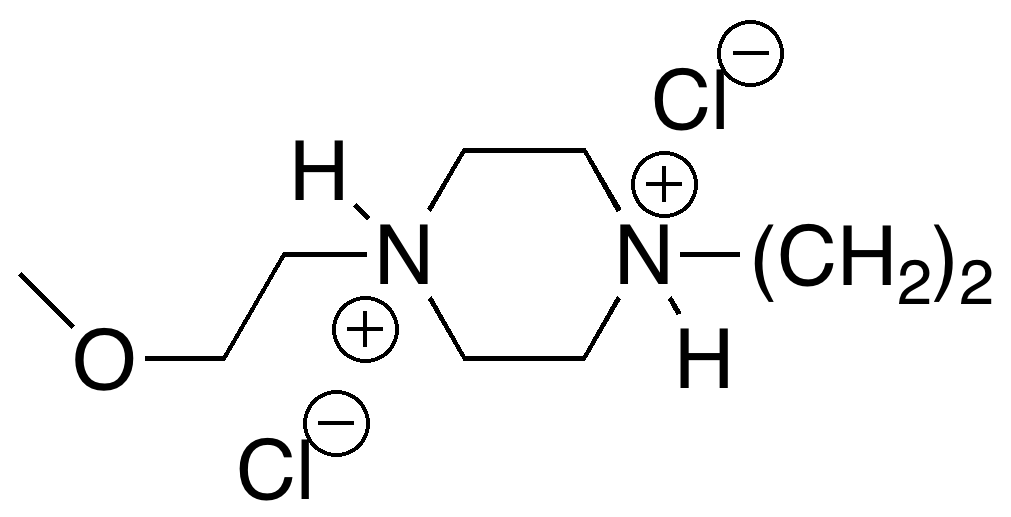** | H | H | >10 |  |
| **8** | NO | Br | H | H | H | **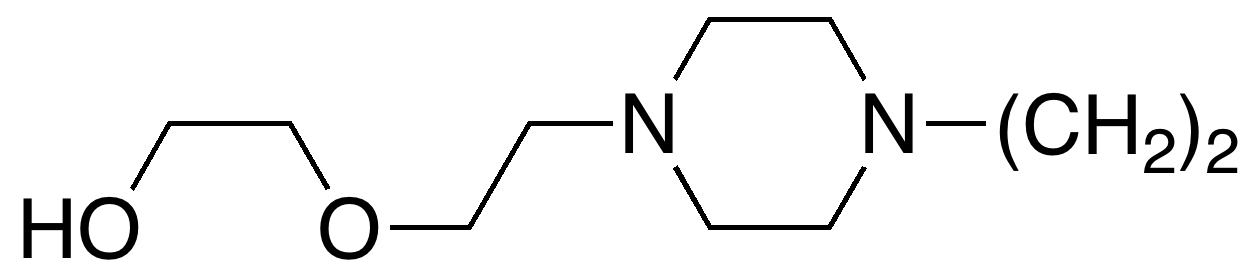** | H | H | >10 |  |
| **9** | NO | Br | H | H | H | **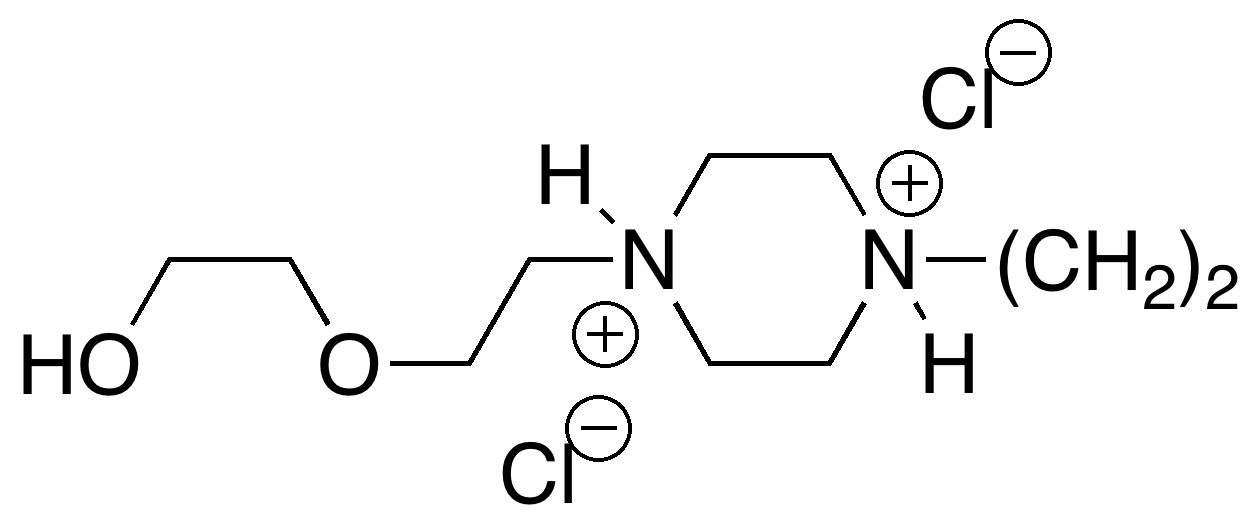** | H | H | >10 |  |
| **10** | NO | Br | H | H | H | **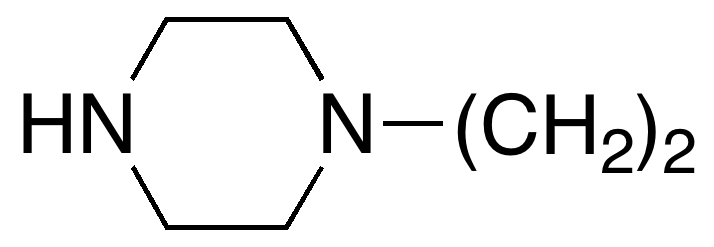** | H | H | 0.7 (± 0.2) |  |
| **11** | NO | Br | H | H | H | 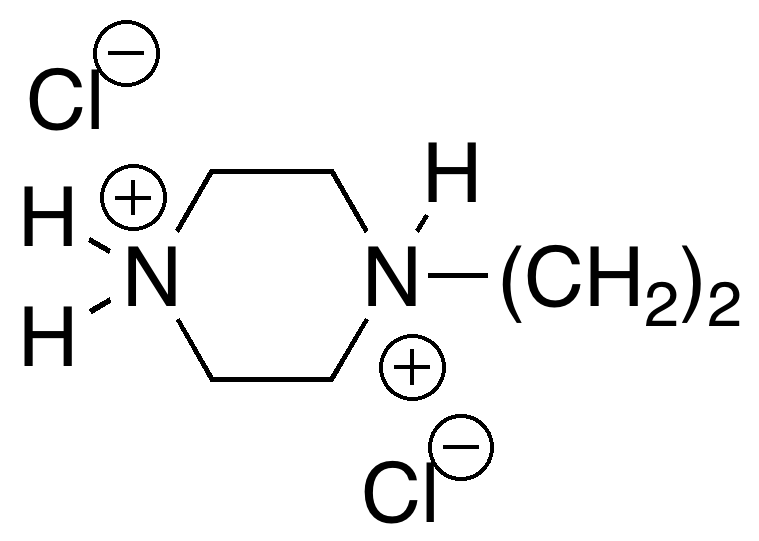 | H | H | 0.25 (± 0.05) |  |
| **12** | NO | Br | H | H | H | 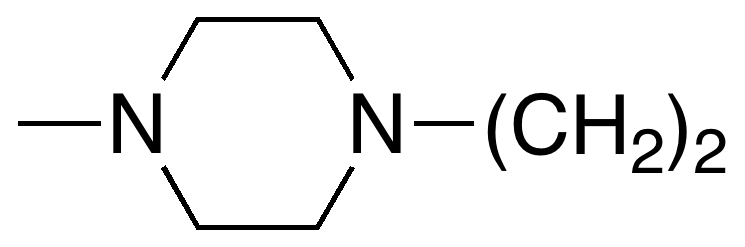 | H | H | 0.49 (± 0.2) |  |
| **13** | NO | Br | H | H | H | 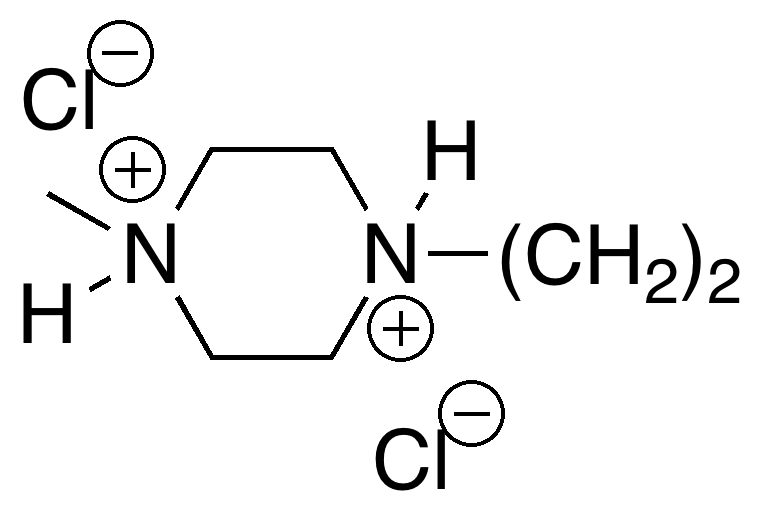 | H | H | 0.45 (± 0.16) |  |
| **14** | NO | Br | H | H | H | 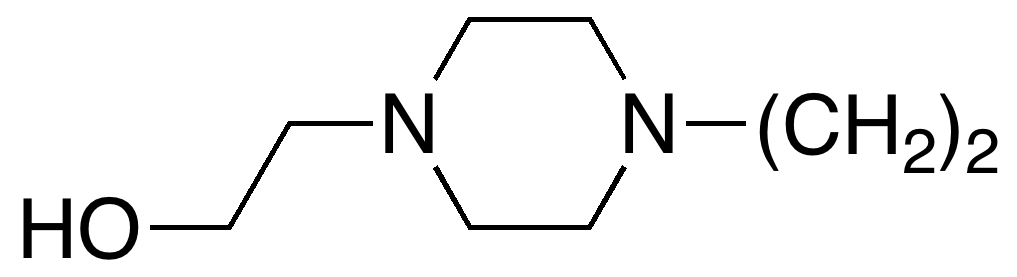 | H | H | >10 |  |
| **15** | NO | Br | H | H | H | 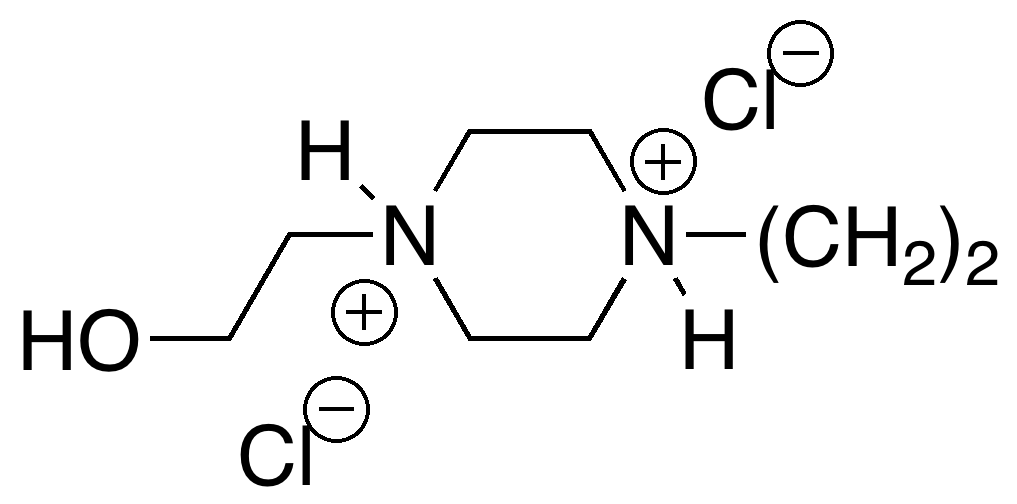 | H | H | >10 |  |
| **16** | NO | Br | H | H | H | 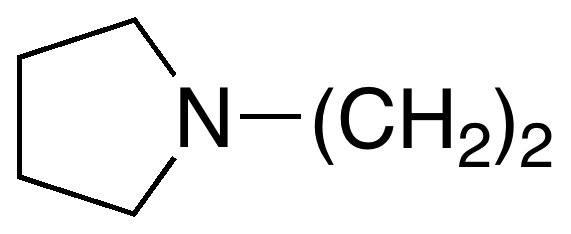 | H | H | >10 |  |
| **17** | NO | Br | H | H | H | 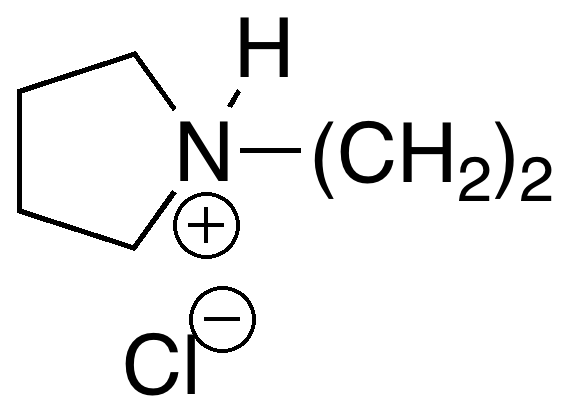 | H | H | 0.44 (± 0.14) |  |
| **18** | NO | Br | H | H | H | 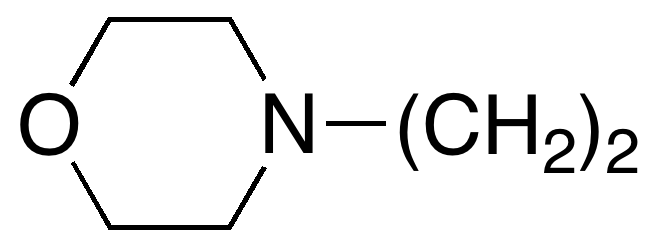 | H | H | >10 |  |
| **19** | NO | Br | H | H | H | 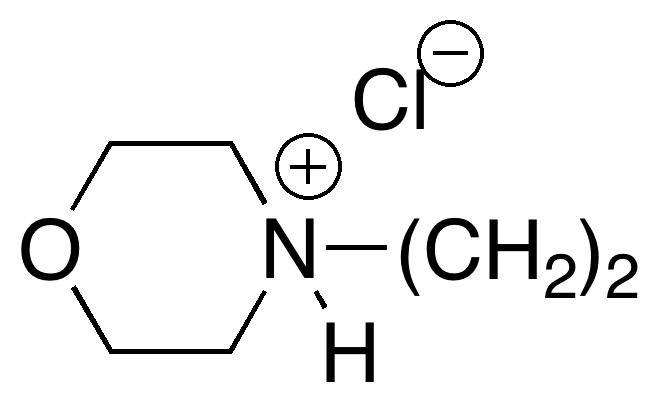 | H | H | >10 |  |
| **20** | O | H | Br | H | H | - | H | H | >10 | **7-substituted** |
| **21** | NO | H | Br | H | H | H | H | H | >10 |  |
| **22** | O | H | Br | COOH | H | - | H | H | >10 |  |
| **23** | NO | H | Br | COOH | H | H | H | H | >10 |  |
| **24** | O | H | Br | H | COOH | - | H | H | >10 |  |
| **25** | NO | H | Br | H | COOMe | H | H | H | >10 |  |
| **26** | NO | H | Br | H | COOH | H | H | H | >10 |  |
| **27** | NO | H | CF_3_ | H | H | H | H | H | >10 |  |
| **28** | NO | H | CF_3_ | COOMe | H | H | H | H | >10 |  |
| **29** | O | H | CF_3_ | COOH | H | - | H | H | >10 |  |
| **30** | NO | H | CF_3_ | COOH | H | H | H | H | >10 |  |
| **31** | O | H | CF_3_ | H | COOH | - | H | H | >10 |  |
| **32** | NO | H | CF_3_ | H | COOMe | H | H | H | 1.30 (± 0.46) |  |
| **33** | NO | H | CF_3_ | H | COOH | H | H | H | >10 |  |
| **34** | O | H | CF_3_ | 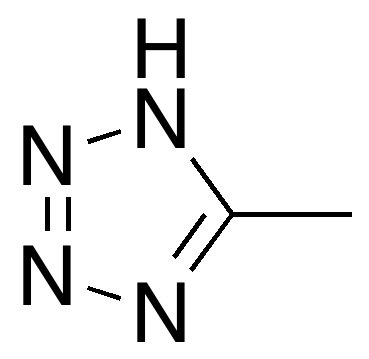 | H | - | H | H | >10 |  |
| **35** | NO | H | CF_3_ | 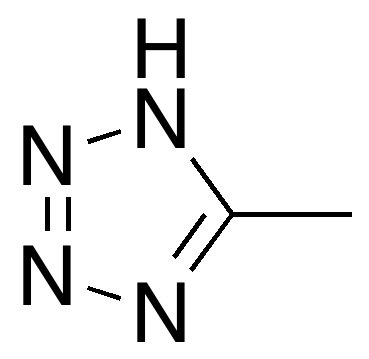 | H | H | H | H | >10 |  |
| **36** | O | H | CF_3_ | H | H | H | H | H | >10 |  |
| **37** | NO | H | CF_3_ | H | H | **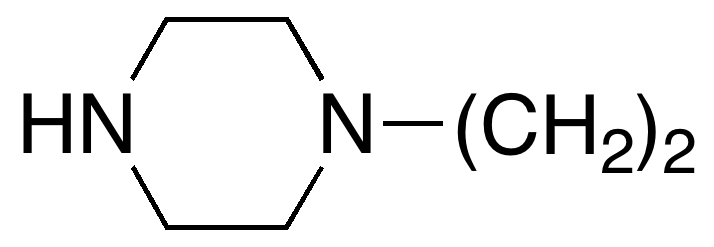** | H | H | >10 |  |
| **38** | NO | H | CF_3_ | COOMe | H | **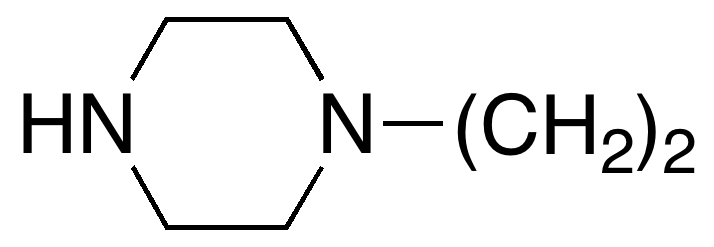** | H | H | >10 |  |
| **39** | NO | H | CF_3_ | COOH | H | **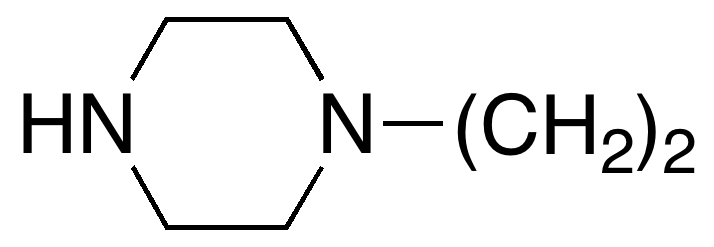** | H | H | >10 |  |
| **40** | NO | H | CF_3_ | H | COOH | **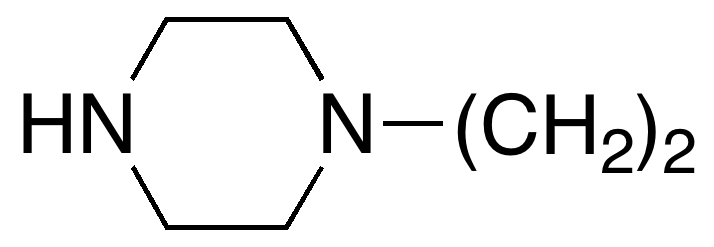** | H | H | >10 |  |
| **41** | NO | H | Br | COOMe | H | **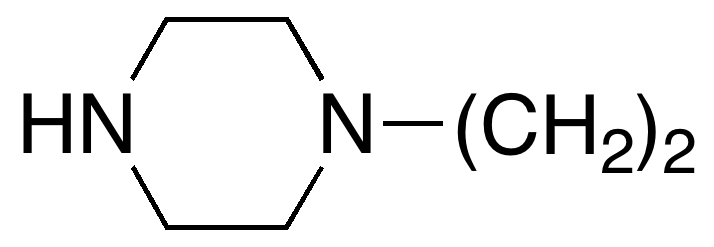** | H | H | >10 |  |
| **42** | NO | H | Br | COOH | H | **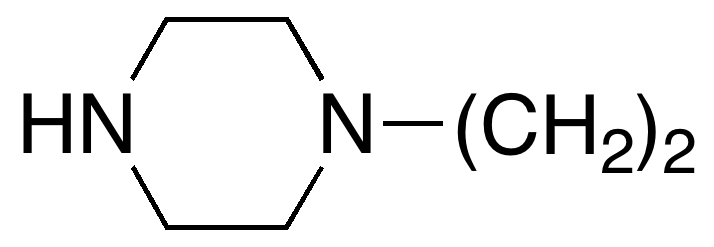** | H | H | >10 |  |
| **43** | NO | H | Br | H | COOMe | **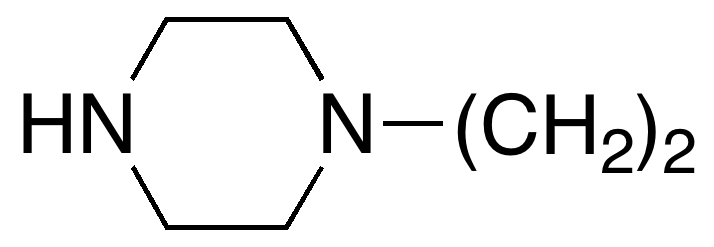** | H | H | >10 |  |
| **44** | NO | H | Br | H | COOH | **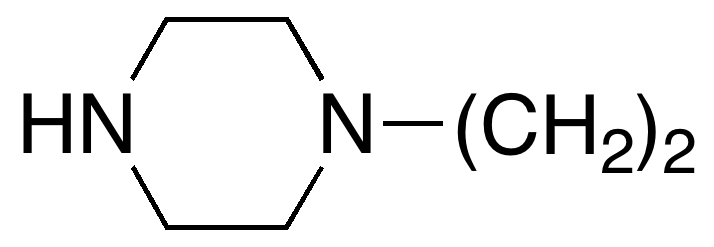** | H | H | >10 |  |
| **45** | NO | 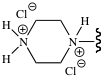 | H | H | H | H | NO_2_ | H | >10 | **6-bulky substituted** |
| **46** | NO | 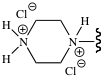 | H | COOH | H | H | NO_2_ | H | >10 |  |
| **47** | NO | **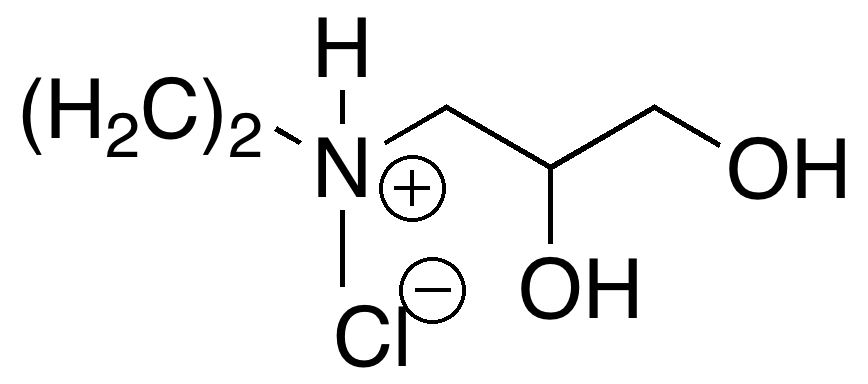** | H | H | H | H | NO_2_ | H | >10 |  |
| **48** | NO | **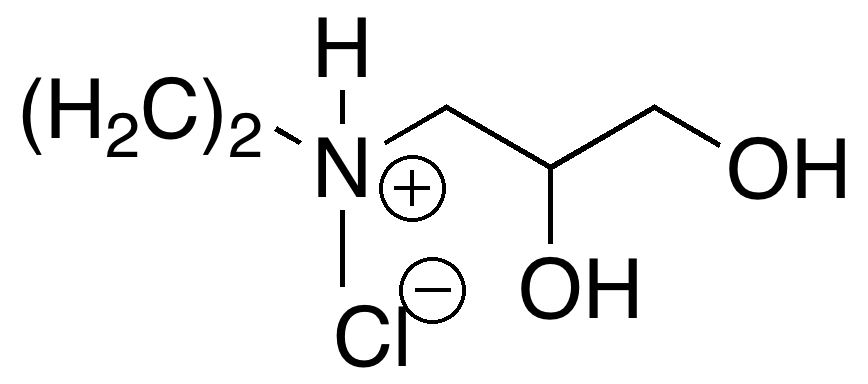** | H | COOH | H | H | NO_2_ | H | >10 |  |
| **49** | NO | **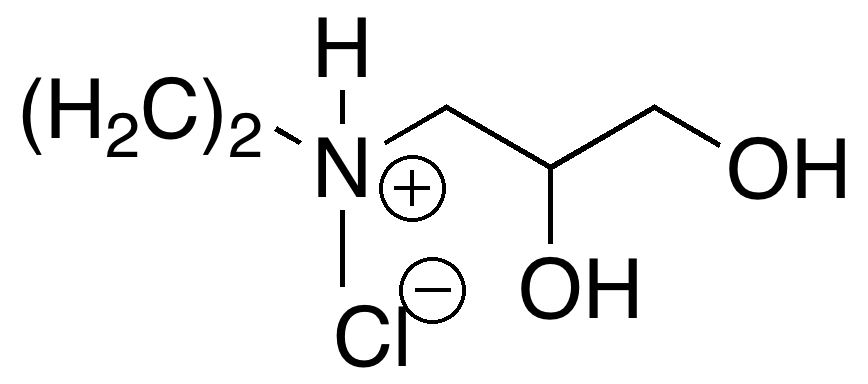** | H | COOMe | H | H | NO_2_ | H | >10 |  |
| **6BIO** | NO | Br | H | H | H | H | H | H | >10 | **6-Br substituted** |
| **5-Me-6-BIO** | NO | Br | H | H | H | H | CH_3_ | H | >10 |  |
| **50** | NO | Br | H | H | H | Ac | H | H | >10 |  |
| **51** | NO | Br | H | H | H | H | NO_2_ | H | >10 |  |
| **52** | NO | Br | H | H | H | H | NH_2_ | H | >10 |  |
| **53** | O | Br | H | H | H | - | NH_2_ | H | >10 |  |
| **54** | O | Br | H | H | H | - | NO_2_ | H | >10 |  |
| **55** | O | Br | H | H | H | - | H | H | >10 |  |
| **56** | NO | Br | H | H | H | NOPO(OEt)_2_ | H | H | >10 |  |
| **57** | O | Br | H | H | H | - | CH_3_ | H | >10 |  |
| **58** | NO | Br | H | H | H | H | H | CH_3_ | >10 |  |
| **59** | NO | Br | H | H | H | Ac | H | CH_3_ | >10 |  |
| **60** | O | H | H | H | H | - | Br | H | >10 | **5 –substituted** |
| **61** | NO | H | H | H | H | H | Br | H | >10 |  |
| **62** | NO | H | H | H | H | H | NH_2_ | H | >10 |  |
| **63** | NO | F | H | H | H | H | H | H | >10 | **6-halogen substituted** |
| **64** | NO | Cl | H | H | H | H | H | H | >10 |  |
| **65** | NO | I | H | H | H | H | H | H | >10 |  |
| **66** | O | H | H | H | H | - | H | H | >10 |  |
| **67** | NO | H | H | H | H | CH_3_ | H | H | >10 |  |
