# Supplementary material for: Indirubin derivatives are potent and selective anti-Trypanosoma cruzi agents
Source: Virulence. 2018 Nov 2;9(1):1658–68. doi: 10.1080/21505594.2018.1532242 (PMC7000199; doi:10.1080/21505594.2018.1532242)
Supplement: Supplemental Material [file kvir-09-01-1532242-g000.zip › Table S2.docx]

**Table S2. Levels of serum and urine toxicity marker enzymes.**

|  |  | **Untreated/Control** | **Treated with vehicle (DMSO/PBS)** | **Treated with Compound 11 (20mg/Kg)** | **Normal values** |
| --- | --- | --- | --- | --- | --- |
| **Blood Serum** | **Creatinine** | 0.375±0.005 | 0.375±0.015 | 0.385±0.015 | **0,2-0,9 mg/dL** |
|  |  |  |  |  |  |
|  | **Aspartate Aminotransferase (AST)** | 132±20 | 177.5±47.5 | 135.5±10.5 | **54-298 (U/I)** |
|  |  |  |  |  |  |
|  | **Alanine Aminotransferase (ALT)** | 31±4 | 47.5±9.5 | 29±2 | **17-77 (U/I)** |
|  |  |  |  |  |  |
|  | **C-Reactive Protein (CRP)** | <0.02 | <0.02 | <0.02 | **<50 mg/dL** |
|  |  |  |  |  |  |
| **Urine (24h)** | **Microalbumin (Urine Albumin)** | 12±3 | 15±2 | 9±1 | **< 20 mg/l** |
|  |  |  |  |  |  |
|  | **Creatinine** | 147±12 | 120±19 | 63±8 | **65,00-148,00 mg/2h** |
|  |  |  |  |  |  |
|  | **Urine albumin to creatinine ratio (ACR)** | 0.08 | 0.125 | 0.142 |  |

Levels of serum and urine toxicity marker enzymes were determined after treatment for 5 consecutive days with compound 11 (20mg/Kg). The analysis was performed in 6 mice per group.
