# Supplementary figures and images for: Indirubin derivatives are potent and selective anti-Trypanosoma cruzi agents
Source: Virulence. 2018 Nov 2;9(1):1658–68. doi: 10.1080/21505594.2018.1532242 (PMC7000199; doi:10.1080/21505594.2018.1532242)

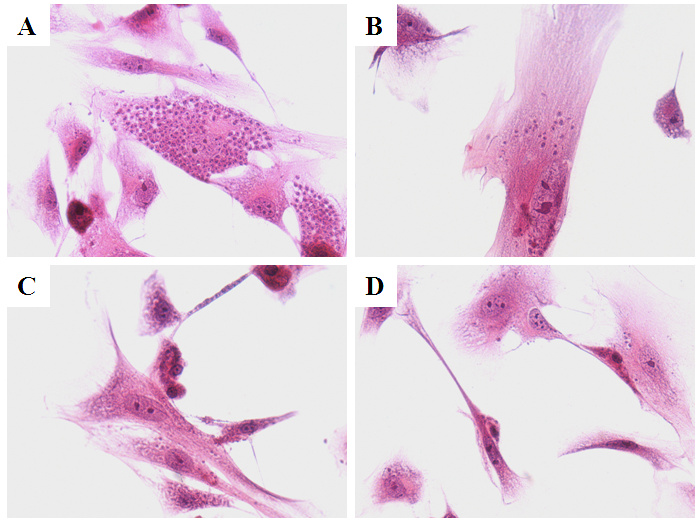

Supplement: Supplemental Material [file kvir-09-01-1532242-g000.zip › Figure S1.tif]
